# Supplementary material for: Nonlocality activation in a photonic quantum network
Source: Nat Commun. 2024 Apr 10;15:3112. doi: 10.1038/s41467-024-47354-w (PMC11006907; doi:10.1038/s41467-024-47354-w)
Supplement: Supplementary file 1 — Supplementary Information [file 41467_2024_47354_MOESM1_ESM.pdf]

Supplementary Information: Nonlocality activation in a photonic quantum network

Luis Villegas-Aguilar,[1] Emanuele Polino,[1] Farzad Ghafari,[1] Marco Túlio Quintino,[2] Kiarn T. Laverick,[3] Ian R. Berkman,[4] Sven Rogge,[4] Lynden K. Shalm,[5] Nora Tischler,[1,*] Eric G. Cavalcanti,[3,†] Sergei Slussarenko,[1] and Geoff J. Pryde[1]

[1]*Centre for Quantum Dynamics and Centre for Quantum Computation and Communication Technology, Griffith University, Yuggera Country, Brisbane, QLD 4111, Australia*
[2]*Sorbonne Université, CNRS, LIP6, Paris F-75005, France*
[3]*Centre for Quantum Dynamics, Griffith University, Yugambeh Country, Gold Coast, QLD 4222, Australia*
[4]*Centre for Quantum Computation and Communication Technology, School of Physics, The University of New South Wales, Sydney, NSW 2052, Australia*
[5]*National Institute of Standards and Technology, 325 Broadway, Boulder, Colorado 80305, USA*

CONTENTS

Supplementary Note 1: Evidence for the Bell-locality of the experimental states 2
Supplementary Note 2: Proving the existence of an LHV model for $(\mathbb{I} \otimes \boldsymbol{\Lambda})(\boldsymbol{\rho})$ 3
Supplementary Note 3: Experimental no-signalling conditions 4
Supplementary References 4

Supplementary Tab. 1 | Experimental results for nonlocality activation for different quantum states. Data for the parameter α of the ideal isotropic state W_α (with which the fidelity of the experimental state is maximized), the corresponding fidelity $\mathcal{F}$, the broadcast inequality $\mathcal{I}_B$, and locality certificate η for our six experimentally prepared states. The fidelity is defined as $\mathcal{F}(\rho_{\text{exp}}, W_\alpha) = \text{Tr}\left(\sqrt{\sqrt{\rho_{\text{exp}}} W_\alpha \sqrt{\rho_{\text{exp}}}}\right)^2$. Green background (**b**) highlights the data for conclusive activation. Yellow background (**c** and **d**) indicates the cases with supporting evidence for activation, but inconclusive locality test results in η.

	α	$\mathcal{F}(\rho_{\text{exp}}, W_\alpha)$	$\mathcal{I}_B$	η
**a**	0.423 ± 0.003	0.9974 ± 0.0004	2.84 ± 0.15	1.49 ± 0.02
**b**	**0.637 ± 0.004**	**0.995 ± 0.003**	**4.24 ± 0.09**	**1.014 ± 0.007**
**c**	0.661 ± 0.003	0.997 ± 0.002	4.27 ± 0.11	0.997 ± 0.006
**d**	0.675 ± 0.004	0.997 ± 0.003	4.34 ± 0.15	0.972 ± 0.006
**e**	0.726 ± 0.008	0.993 ± 0.003	4.83 ± 0.18	0.89 ± 0.01
**f**	0.862 ± 0.008	0.991 ± 0.006	5.69 ± 0.19	0.775 ± 0.006

* n.tischler@griffith.edu.au
† e.cavalcanti@griffith.edu.au

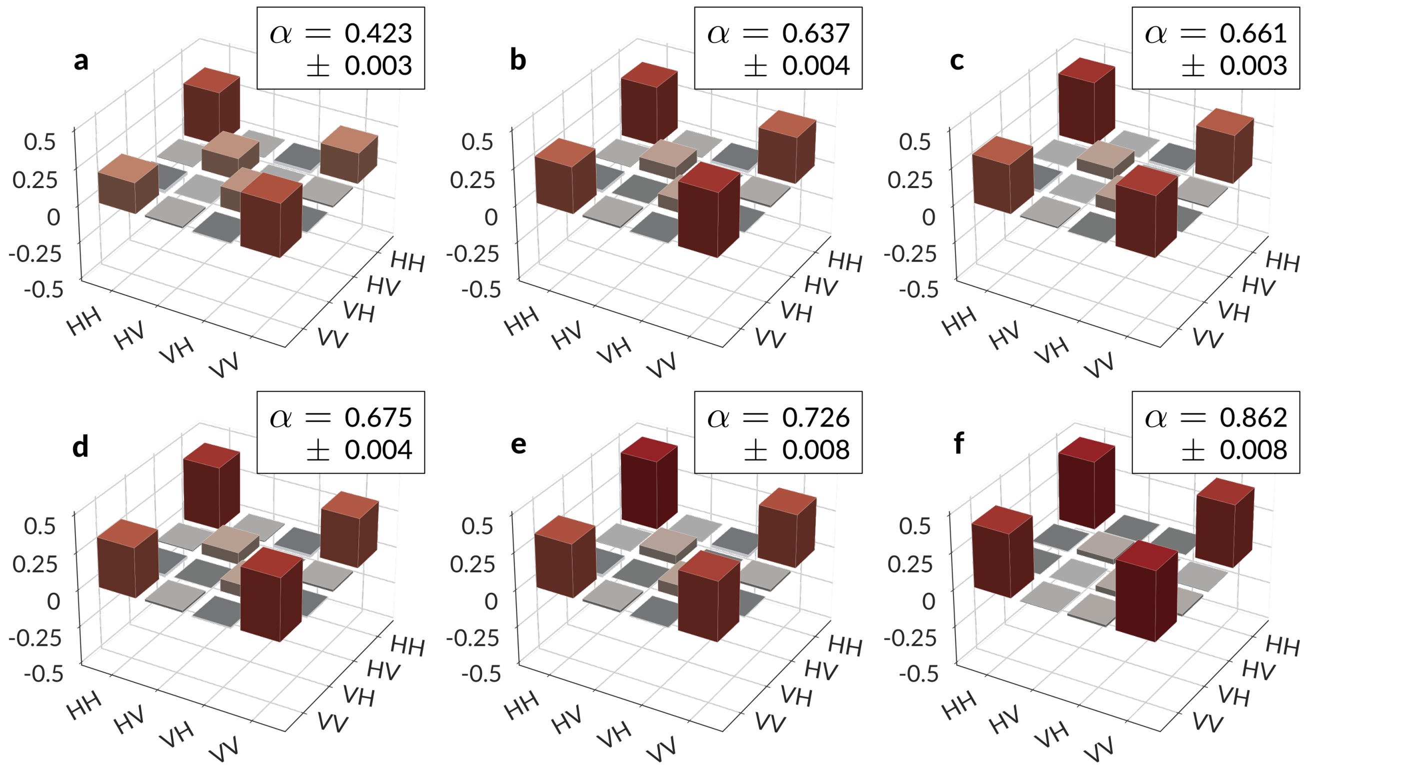

Supplementary Fig. 1 | Experimental quantum states. **a** Real part of the density matrix ρ_{exp}, reconstructed through quantum state tomography, for $\alpha = 0.423 \pm 0.003$. **b–f** Same as **a**, but for $\alpha = 0.637 \pm 0.004$, $\alpha = 0.661 \pm 0.003$, $\alpha = 0.675 \pm 0.004$, $\alpha = 0.726 \pm 0.008$, and $\alpha = 0.862 \pm 0.008$, respectively. The absolute values of the imaginary parts were all below 0.02, except for **e**, which were below 0.04.

Supplementary Note 1: Evidence for the Bell-locality of the experimental states

For each experimental density matrix, we generated a large ensemble of associated states in a Monte Carlo simulation, which included systematic and statistical errors. We used these ensembles to certify the locality of the original states with statistical significance. We subjected all of the derived states to our locality testing procedure described in the main text. For the experimental state ρ_{exp} with an associated $\alpha = 0.637 \pm 0.004$ parameter (red triangle in Figure 3), we obtained a value $\eta \geq 1$ for 98.1% of all sampled density matrices. This result implies a low probability of erroneously classifying ρ_{exp} as Bell-local. For the $\alpha = 0.661 \pm 0.003$ state, 16.8% matrices produced a local result.

As mentioned in the main text, a $\eta < 1$ value does not imply that ρ_{exp} is Bell-nonlocal. We further analyzed the bipartite nonlocality of the six experimental states (and associated Monte Carlo distributions) via the Horodecki criterion [1], which is a sufficient condition for Bell-nonlocality for general mixed two-qubit states. It provides the maximum value possible for the CHSH [2] inequality under projective measurements. It is possible to associate a correlation matrix T_ρ to any two-qubit state ρ. This correlation matrix has entries $t_{ij} = \text{Tr}\left[\rho(\sigma_i \otimes \sigma_j)\right]$ for $i, j = 1, 2, 3$, where σ_i represent the standard Pauli matrices. The Horodecki criterion then gives the maximum possible CHSH value B for a given state ρ:

$$\max\langle B\rangle_\rho = 2\sqrt{m_{11}^2 + m_{22}^2} \geq 2, \tag{1}$$

where m_{11}^2 and m_{22}^2 denote the two largest eigenvalues of $T_\rho T_\rho^T$.

These results are presented as a violin plot in Supplementary Fig. 2, which provides an intuitive way to

visualize the results for the distribution in the calculated values of $\max\langle B\rangle_\rho$. For all experimental states ρ_{exp} with $\alpha < 0.726$, we obtained a value $\max\langle B\rangle_\rho < 2$ for 100%of the simulated density matrices. It is important to acknowledge that although $\max\langle B\rangle_\rho \geq 2$ provides definitive evidence of Bell nonlocality for the sampled states, a value below this bound does not guarantee Bell locality. These states, in principle, may still have the potential to violate other bipartite Bell inequalities. This is the reason why the two experimental states ρ_{exp} that violate the broadcast inequality but do not have a conclusive certificate parameter $\eta > 1$ (red dots in Figure 3 and Figure 4) are depicted in the region of uncertainty for activation in Figure 4 of the main text.

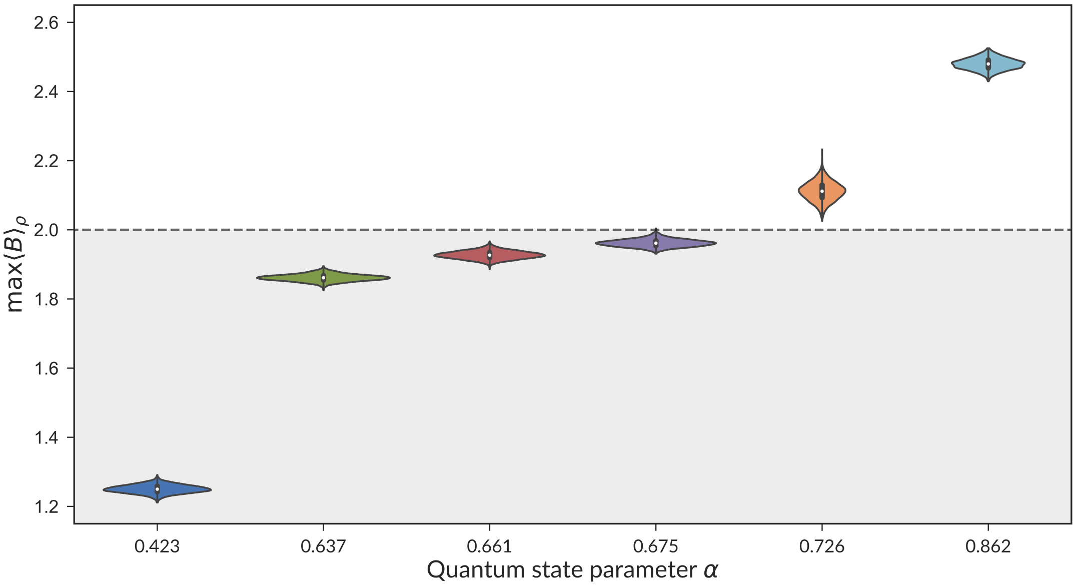

Supplementary Fig. 2 | Maximum violation of the CHSH inequality under projective measurements for different experimental states. We simulated 2000 samples of each experimental density matrix via Monte Carlo simulation; the rotated kernel density plots associated with each data point show the results for the derived distributions. The grey area indicates the region where the CHSH inequality is not violated.

Supplementary Note 2: Proving the existence of an LHV model for $(\mathbb{1}\otimes\Lambda)(\rho)$

Here we prove the following statement:

If ρ is a state that admits an LHV model for all n-outcome measurements, and Λ is a positive trace-preserving linear map such that $(\mathbb{1}\otimes\Lambda)(\rho)$ is a quantum state, then $(\mathbb{1}\otimes\Lambda)(\rho)$ also admits an LHV model for all n-outcome measurements.

Since we know that ρ admits an LHV model for n-outcome measurements, then for any n-outcome POVMs $\{A_{a|x}\}$ and $\{B_{b|y}\}$ for Alice and Bob, there exist distributions $\{p(\lambda)\}$, $\{p_{\text{A}}(a|x,\lambda)\}$, and $\{p_{\text{B}}(b|y,\lambda)\}$ such that

$$p(a,b|x,y) = \text{Tr}\left[\rho(A_{a|x}\otimes B_{b|y})\right] = \int d\lambda\, p(\lambda) p_{\text{A}}(a|x,\lambda) p_{\text{B}}(b|y,\lambda)\,. \tag{2}$$

We can now consider the probability distribution for the same measurements with the state $(\mathbb{1}\otimes\Lambda)(\rho)$, i.e.,

$$\begin{aligned} q(a,b|x,y) &= \text{Tr}\left[(\mathbb{1}\otimes\Lambda)(\rho)(A_{a|x}\otimes B_{b|y})\right] \\ &= \text{Tr}\left[\rho(\mathbb{1}\otimes\Lambda)^\dagger(A_{a|x}\otimes B_{b|y})\right] \\ &= \text{Tr}\left[\rho(A_{a|x}\otimes\Lambda^\dagger(B_{b|y}))\right]. \end{aligned} \tag{3}$$

where for the sake of clarity, we are using q to denote the probabilities resulting from state $(\mathbb{I} \otimes \Lambda)(\rho)$. Since Λ is a positive trace-preserving map, its adjoint is necessarily a positive unital map. This means that $\{\Lambda^\dagger(B_{b|y})\}$ is also an n-outcome POVM. Denoting $\Lambda^\dagger(B_{b|y}) = B'_{b|y}$, we have

$$q(a, b|x, y) = \mathrm{Tr}[\rho(A_{a|x} \otimes B'_{b|y})] = \int d\lambda\, p(\lambda) p_{\mathrm{A}}(a|x, \lambda) p_{\mathrm{B}}(b|y, \lambda), \tag{4}$$

where the final equality results from equation (2) since it holds for any valid pair of POVMs.

Supplementary Note 3: Experimental no-signalling conditions

Experimental probabilities are derived from a finite number of samples, introducing unavoidable statistical fluctuations. Consequently, any non-signalling constraint can only be approximately satisfied, even in the case of space-like separation between parties. We explicitly verified that our results are consistent with the no-signalling condition formalized by equations (7) and (8) in the Methods. To check this, we computed the expressions:

$$E_{\mathrm{B}}^{\mathrm{NS}}(y, y', z, c) = \left| \sum_b p_{\mathrm{BC}}(b, c|y, z) - p_{\mathrm{BC}}(b, c|y', z) \right| \quad \forall\, y, y', z, c, \tag{5}$$

$$E_{\mathrm{C}}^{\mathrm{NS}}(y, z, z', b) = \left| \sum_c p_{\mathrm{BC}}(b, c|y, z) - p_{\mathrm{BC}}(b, c|y, z') \right| \quad \forall\, y, z, z', b. \tag{6}$$

To satisfy the no-signalling condition, it follows that $E_{\mathrm{B}}^{\mathrm{NS}} = E_{\mathrm{C}}^{\mathrm{NS}} = 0$. We computed the expressions (5) and (6) for all non-trivial combinations ($y \neq y'$, $z \neq z'$). As shown in Supplementary Tab. **2**, we found the mean value of these expressions to be $\langle E^{\mathrm{NS}} \rangle = 0.02 \pm 0.05$, indicating that our results are consistent with no-signalling being satisfied.

Supplementary Tab. 2 | No-signalling condition between Bob and Charlie

z	$E_{\mathrm{B}}^{\mathrm{NS}}(y \neq y')$	y	$E_{\mathrm{C}}^{\mathrm{NS}}(z \neq z')$
0	1.66e-02 ± 4.37e-02	0	3.75e-02 ± 4.17e-02
1	2.96e-04 ± 4.32e-02	1	1.63e-02 ± 4.74e-02

Supplementary References

[1] Horodecki, R., Horodecki, P. & Horodecki, M. Violating Bell inequality by mixed spin-12 states: Necessary and sufficient condition. *Phys. Lett. A* **200**, 340–344 (1995).
[2] Clauser, J. F., Horne, M. A., Shimony, A. & Holt, R. A. Proposed experiment to test local hidden-variable theories. *Phys. Rev. Lett.* **23**, 880–884 (1969).
